# Maturation of *Streptococcus pneumoniae* lipoproteins by a type II signal peptidase is required for ABC transporter function and full virulence

**DOI:** 10.1111/j.1365-2958.2007.06065.x

**Published:** 2007-12-16

**Authors:** Suneeta Khandavilli, Karen A Homer, Jose Yuste, Shilpa Basavanna, Timothy Mitchell, Jeremy S Brown

**Affiliations:** 1 Centre for Respiratory Research, Department of Medicine, Royal Free and University College Medical School, Rayne Institute London WC1E 6JJ, UK; 2 Department of Microbiology, King's College London, Dental Institute London SE1 9RT, UK; 3 Division of Infection and Immunity, IBLS, University of Glasgow Glasgow, G12 8TA, UK

## Abstract

Cell surface lipoproteins are important for the full virulence of several bacterial pathogens, including *Streptococcus pneumoniae*. Processing of prolipoproteins seems to be conserved among different bacterial species, and requires type II signal peptidase (Lsp) mediated cleavage of the N-terminal signal peptide to form the mature lipoprotein. Lsp has been suggested as a target for new antibiotic therapies, but at present there are only limited data on the function of Lsp for Gram-positive bacterial pathogens. We have investigated the function and role during disease pathogenesis of the *S. pneumoniae* Lsp, which, blast searches suggest, is encoded by the gene Sp0928. Expression of Sp0928 protected *Escherichia coli* against the Lsp antagonist globomycin, and proteomics and immunoblot analysis demonstrated that deletion of Sp0928 prevented processing of *S. pneumoniae* prolipoproteins to mature lipoproteins. These data strongly suggest that Sp0928 encodes the *S. pneumoniae* Lsp. However, immunoblots of membrane-associated proteins, immunoelectron microscopy and flow cytometry assays all confirmed that in the absence of Lsp, immature lipoproteins were still attached to the cell surface. Despite preservation of lipoprotein attachment to the cell membrane, loss of *S. pneumoniae* Lsp resulted in several phenotypes associated with impaired lipoprotein function and reduced *S. pneumoniae* replication in animal models of infection.

## Introduction

Bacterial surface proteins are important for the interaction of bacterial pathogens with their environment during infection and, as a consequence, are frequently involved in disease pathogenesis. One large category of bacterial surface proteins is the lipoproteins. Lipoproteins are important components of ABC transporters, transmembrane structures involved in the import or export of a wide range of substrates, including sugars, amino acids, oligopeptides, polyamines, various metal ions and minerals (Garmory and Titball, 2004). ABC transporters contribute to many bacterial processes, such as acquisition of vital nutrients, stress responses and intercellular signalling, many of which could be vital for bacterial growth and survival within the host. Signature-tagged mutagenesis (STM) screens for virulence genes and detailed investigations of individual ABC transporters have confirmed that ABC transporters do indeed have significant roles during disease pathogenesis for a range of bacteria, including the important Gram-positive respiratory and systemic pathogen *Streptococcus pneumoniae* (Mei *et al*., 1997; Chiang and Mekalanos, 1998; Polissi *et al*., 1998; Lau *et al*., 2001; Hava and Camilli, 2002). *S. pneumoniae* ABC transporters that affect interactions with the host include the pneumococcal surface adhesin A (PsaA), a manganese uptake transporter required for resistance to oxidative stress and adherence to host cells (Dintilhac *et al*., 1997; Marra *et al*., 2002), the Piu, Pit and Pia iron uptake transporters (Brown *et al*., 2001a; 2002), the Ami–AliA/B oliogpeptide transporters (Kerr *et al*., 2004) and several uncharacterized ABC transporters identified by STM screens (Polissi *et al*., 1998; Lau *et al*., 2001; Hava and Camilli, 2002). As well as lipoproteins associated with ABC transporter function, the *S. pneumoniae* genome also encodes at least 15 other lipoproteins (Tettelin *et al*., 2001). The functions of many of these non-ABC transporter lipoproteins are unknown, but two, PpmA (putative proteinase maturation protein A) and SlrA (streptococcal lipoprotein rotamase A), belong to the chaperone family of peptidyl-prolyl isomerases and contribute to *S. pneumoniae* colonization, avoidance of phagocytosis and pulmonary infection (Overweg *et al*., 2000; Hermans *et al*., 2006).

Lipoproteins are covalently attached to the extracellular surface of the cell membrane by a mechanism that seems to be largely conserved among eubacteria (Sutcliffe and Harrington, 2002). Initially, prolipoproteins are secreted out of the cell by the general secretory pathway, and this is directed by an N-terminal signal peptide sequence. After secretion, lipoproteins are covalently linked to the cell membrane by the enzyme diacylglyceryl transferase (Lgt), which catalyses the attachment of the thiol of a universally conserved cysteine residue within the ‘lipobox’ domain of the lipoprotein signal peptide to membrane phospholipid diacylglycerol (Tokunaga *et al*., 1982; Inouye *et al*., 1983; Sutcliffe and Harrington, 2002). A type II lipoprotein signal peptidase (Lsp) then cleaves the N-terminal signal peptide adjacent to the ‘lipobox’ cysteine residue to form the mature lipoprotein. In Gram-negative bacteria, the mature lipoprotein undergoes additional modification by attachment of an amide-linked fatty acid to the N-terminal cysteine residue of the lipoprotein by an N-acyl transferase (Lnt), but an equivalent enzyme is not found in Gram-positive genomes (Sankaran and Wu, 1994; Tjalsma *et al*., 1999a; Sutcliffe and Harrington, 2002).

The importance of lipoproteins for disease pathogenesis suggests that inhibition of lipoprotein processing by Lsp may strongly affect bacterial virulence. However, despite the conservation of the pathway for processing lipoproteins, the available data on the effects of inhibition of Lsp function indicate that these are surprisingly variable between bacterial species. For example, inhibition of Lsp is toxic to *Escherichia coli*, yet only affects growth of *Bacillus subtilis* under stress conditions (Inouye *et al*., 1983; Tjalsma *et al*., 1999a). At present, there are no published data on Lsp function in *S. pneumoniae*, and only limited data for other Gram-positive pathogens, with reports available on the effects of deletion or disruption of *lsp* for *Mycobacterium tuberculosis*, *Listeria monocytogenes* and *Streptococcus suis* (De Greef *et al*., 2003; Reglier-Poupet *et al*., 2003; Sander *et al*., 2004). Disruption of *lsp* in *M. tuberculosis* had no detectable effect on growth or cell morphology, but did result in the accumulation of unprocessed forms of the two lipoproteins investigated (Sander *et al*., 2004). Similarly, disruption of *lsp* in *L. monocytogenes* and *S. suis* resulted in the retention of the N-terminal signal peptides for the limited number of lipoproteins investigated (De Greef *et al*., 2003; Reglier-Poupet *et al*., 2003). However, the effects of loss of Lsp on virulence varied between species, with a strong effect on *M. tuberculosis*, impaired intracellular growth and moderately attenuated virulence for *L. monocytogenes*, but no effect on the virulence of *S. suis* (De Greef *et al*., 2003; Reglier-Poupet *et al*., 2003; Sander *et al*., 2004). STM screens have also identified *lsp* as probably required for *Staphylococcus aureus* virulence (Mei *et al*., 1997). In addition, disruption of *lgt* in different bacterial species has a variable effect on virulence, rendering *S. pneumoniae* avirulent in a mouse model of pneumonia, only partially attenuating the virulence of *Streptococcus equi* and actually increasing the virulence of *S. aureus* (Petit *et al*., 2001; Hamilton *et al*., 2006; Wardenburg *et al*., 2006).

The *S. pneumoniae* genome contains a putative four-gene operon (gene numbers Sp0927–0930 in the TIGR4 *S. pneumoniae* genome), one of which (Sp0928), blast searches suggest, is likely to encode the *S. pneumoniae* Lsp (Tettelin *et al*., 2001). Two *S. pneumoniae* STM screens independently identified mutants containing insertions in the first gene of this putative operon, indicating that this operon is likely to be important for disease pathogenesis (Lau *et al*., 2001; Hava and Camilli, 2002). However, the Sp0927–0930 operon also contains genes encoding CbpE (Sp0930), a choline-binding protein involved with processing cell wall phosphorylcholine (Hermoso *et al*., 2005), and a LysR regulator (Sp0927), loss of function of which might also affect virulence, and why this putative operon is required for disease pathogenesis needs further evaluation. In this study, we have investigated the function of the product of *S. pneumoniae* Sp0928, characterizing its potential role as an Lsp and for lipoprotein function, and assessing its importance during disease pathogenesis in mouse models of *S. pneumoniae* infection.

## Results

### Genetic organization of the Sp0927–0930 operon and creation of an Sp0928 deletion mutant

**Fig. 1:**
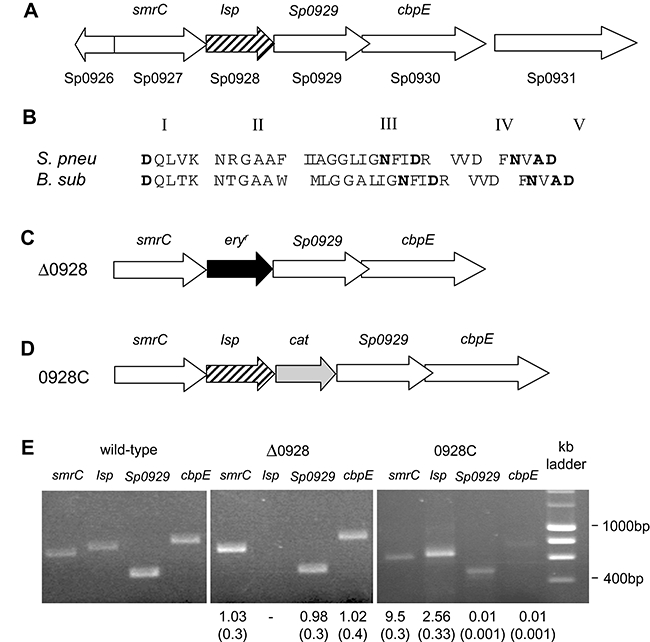
A. Organization of the Sp0927–0930 loci, with assigned gene names above the arrows and the TIGR4 genome gene number below the line. Arrows indicate transcriptional direction, and the putative *lsp* gene is shaded with diagonal lines. B. Alignment of the derived protein sequence of Sp0928 (*S. pneu*) to the conserved domains of the *B. subtilis* (*B. sub*) Lsp (Tjalsma *et al*., 1999). The six residues required for Lsp activity are marked in bold, and are 100% conserved between the *B. subtilis* Lsp and the protein encoded by SP0928. C. Structure of the Sp0927–930 locus in the Δ0928 mutant strain derived from the wild-type 0100993 strain, showing replacement of Sp0928 with an in-frame copy of *ery*^r^. D. Structure of the Sp0927–930 locus in the 0928C mutant strain derived from the Δ0928 mutant strain, showing replacement of the *ery*^r^ cassette with an in-frame copy of Sp0928 (*lsp*) and *cat*. E. Ethidium bromide stained agarose gels showing RT-PCR analysis of the Sp0927–0930 locus in the wild-type, Δ0928 and 0928C strains. Primers were designed to amplify internal portions of each gene within the locus, using the following primer pairs: Sp0927F and Ery-Sp0927R for Sp0927; Sp0928F and Sp0928R for Sp0928; Ery-Sp0929F and Sp0929R for Sp0929; and Sp0930F and Sp0930R for Sp0939. Reactions not containing reverse transcriptase gave no products (not shown). The mean (SD in brackets) relative transcription of each gene in the mutant strains compared with the wild-type strain (obtained using real-time RT-PCR) is stated below the corresponding band in the gel. No product was obtained for Sp0928 in the Δ0928 mutant strain.

The Sp0927–0930 locus of the *S. pneumoniae* TIGR4 genome consists of four genes with overlapping open reading frames (ORFs) that are transcribed in the same direction (Fig. 1A). Reverse transcriptase polymerase chain reaction (RT-PCR) analysis of this locus using primers designed to span the junctions of each gene confirmed that all four genes are co-transcribed as a single transcript that terminates after Sp0930 (data not shown). blast searches show that the protein encoded by the second gene in this operon, Sp0928, has a high degree of identity and similarity to proven and putative Lsp enzymes of other bacteria (Table 1), and Sp0928 has been annotated as *lsp* in the TIGR4 genome sequence (Tettelin *et al*., 2001). Analysis using hidden Markoff Models and HMMTOP software (Tusnady and Simon, 1998) predicts that the sequence of the protein product of Sp0928 has four transmembrane domains and two extracellular loops as suggested for other Lsp proteins(Pragai *et al*., 1997; Tjalsma *et al*., 1999b). In addition, the Sp0928 product contains domains with a high degree of identity to the *B. subtilis* Lsp conserved regions I to V, including conservation of all of the six amino acids that are important for Lsp activity (Fig. 1B; Tjalsma *et al*., 1999b). The other proteins encoded by the genes within this operon are a putative LysR regulator (Sp0927), a pseudouridine synthase (Sp0929), and the choline-binding protein termed CbpE that is involved in the processing of cell wall phosphorylcholine and adhesion to host cells (Sp0930; Hermoso *et al*., 2005). There are no published data on the function of Sp0927, Sp0928 and Sp0929, although STM screens have identified strains with polar mutations in Sp0927 as attenuated in virulence (Lau *et al*., 2001; Hava and Camilli, 2002). The Sp0927–0930 locus is conserved among the sequenced *S. pneumoniae* genomes, and as previously reported, homologues of Sp0927, Sp0928 and Sp0929 are organized as probable operons in other streptococcal species (De Greeff *et al*., 2003).

**Table 1:** Identity and similarity of the predicted amino acid sequence of Sp0928 to the predicted amino acid sequences of Lsp for other bacterial species.

Organism	Gene number	% identity/similarity	Length of amino acids compared
*Streptococcus pyogenes*	Spy_0637	64/76	137
*Streptococcus mutans*	SMU.853	62/79	138
*S. suis*	SSU05	55/73	138
*Lactococcus lactis*	Llmg_1525	51/71	132
*B. subtilus*	BSU15450	38/59	131
*L. monocytogenes*	LMRG_00991	38/59	135
*S. aureus*	SAB1060	38/63	136
*M. tuberculosis*	TBFG_11572	34/53	133
*E. coli*	ECP_0025	34/54	128

To study the role of Lsp in *S. pneumoniae*, an isogenic non-polar deletion mutant (Δ0928) with the Sp0928 gene replaced by an erythromycin resistance cassette (*erm*) was constructed using overlap extension PCR (Fig. 1C). An additional strain was constructed in which the Δ0928 strain was complemented by replacement of the *erm* cassette with an in-frame copy of Sp0928 combined with the chloramphenicol-resistant marker *cat* (termed 0928C; Fig. 1D). RT-PCR analysis of the mutant strains demonstrated that the Sp0928 product was absent in the Δ0928 strain but was present in the wild-type and 0928C strains, and confirmed that the mutations affecting Sp0928 were non-polar and did not prevent transcription of Sp0929 and Sp0930 (Fig. 1E). No products were obtained for RT-PCR reactions that did not contain reverse transcriptase, demonstrating that contaminating DNA was not responsible for the results obtained. As non-quantitative RT-PCR suggested there was some dysregulation of gene expression in the 0928C strain (Fig. 1E), the relative levels of transcripts for all three strains were compared using real-time RT-PCR (Fig. 1E). The results show that in the Δ0928 strain, transcription of Sp0927, Sp0929 and Sp0930 was not significantly affected. However, transcription of all four genes was significantly disrupted in the complemented strain, with increased expression of Sp0927 and Sp0928 and decreased expression of Sp0929 and Sp0930 compared with the wild-type and Δ0928 strains.

### Sp0928 antagonises globomycin toxicity in *E. coli*

**Fig. 2:**
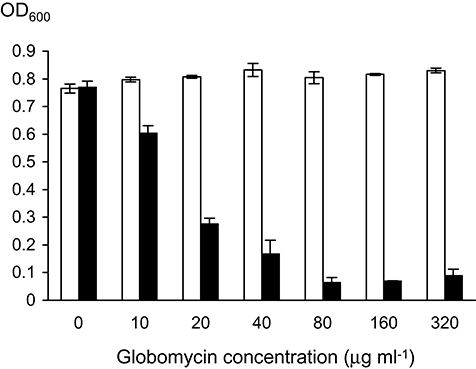
Globomycin-resistance assay. Resistance of *E. coli* expressing full-length Sp0928 (clear columns) or a lipoprotein (Sp0149, black columns) to increasing concentrations of globomycin, measured by OD_600_ after overnight culture in LB broth at 37°C. For the comparison of the results between Sp0928 and Sp0149, *P* < 0.001 at all concentrations of globomycin (Student's *t*-test).

The antibiotic globomycin specifically inhibits Lsp function (Inukai *et al*., 1978) and is toxic to *E. coli*, probably because of the accumulation of pro-lipoproteins in the inner membrane. Both Gram-negative and Gram-positive bacterial *lsp* genes expressed in *E. coli* antagonize globomycin toxicity, and therefore confer resistance to this antibiotic, and this property has been used widely to demonstrate Lsp activity for proteins encoded by putative *lsp* genes (Pragai *et al*., 1997; De Greeff *et al*., 2003; Rahman *et al*., 2007). Hence, in order to demonstrate that Sp0928 encodes an Lsp, the full-length Sp0928 gene was ligated into the expression vector pQE30UA and transformed into *E. coli*. As indicated by optical density (OD) measurements (Fig. 2), *E. coli* expressing *S. pneumoniae* Sp0928 grew well in the presence of increasing concentrations of globomycin up to 320 μg ml^−1^. In contrast, growth of a negative control *E. coli* strain expressing a *S. pneumoniae* lipoprotein (Sp0149, S. Basavanna and J. Brown, unpubl. data) was inhibited at all the concentrations of globomycin used. These results strongly suggest that Sp0928 does encode an Lsp enzyme and can be termed *lsp*.

### Loss of Sp0928 results in increased lipoprotein size compatible with retention of the N-terminal signal peptide

**Fig. 3:**
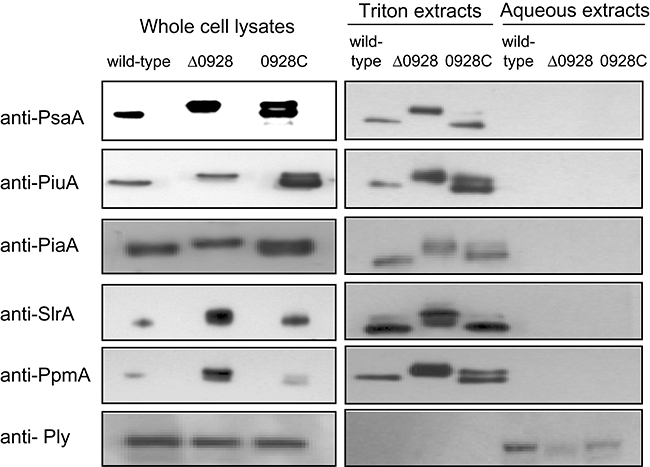
Immunoblots using antibodies to *S. pneumoniae* lipoproteins (PsaA, PiuA, PiaA, SlrA and PpmA) and pneumolysin (Ply) to probe whole-cell lysates and the lipid and aqueous phases of Triton X-114 extracts of wild-type, Δ0928 and 0928C bacteria. Sizes of lipoproteins were approximately 1–2 kDa larger in the Δ0928 strain, and in the wild-type strain were similar to the purified recombinant lipoprotein without the N-terminal signal peptide sequence for PiuA and PiaA (data not shown).

In order to assess the effect of deletion of *lsp* on the processing of *S. pneumoniae* lipoproteins, Western blots of whole-cell lysates of the wild-type and the Δ0928 and 0928C mutant strains were probed with polyclonal mouse or rabbit antibodies to three lipoproteins (PiuA, PiaA and PsaA) that are constituents of metal ion uptake ABC transporters, and two lipoproteins that are not associated with ABC transporters (SlrA and PpmA) (Dintilhac *et al*., 1997; Brown *et al*., 2001b; Adrian *et al*., 2004; Jomaa *et al*., 2005). For all five antibodies, the main signal for the lipoprotein in the Δ0928 strain had a slightly higher molecular weight compared with the wild-type strain (Fig. 3), compatible with retention of the N-terminal signal peptide sequence which ranges from 18 to 23 amino acids in length for these lipoproteins. When used to probe the Δ0928 strain, antibodies to SlrA and PpmA also seem to result in an additional lower-intensity signal closer in size to the processed lipoprotein, possibly suggesting that for these lipoproteins loss of *lsp* may not completely prevent N signal peptide processing. Immunoblots using an antibody to pneumolysin, a non-lipoprotein *S. pneumoniae* protein, resulted in similar sized bands for the wild-type and Δ0928 strains (Fig. 3), indicating that the increase in protein size in the Δ0928 strain was specific for lipoproteins. In the complemented strain 0928C, immunoblots for lipoproteins identified either a single band of similar size to the band in the wild-type strain or two bands corresponding to the bands seen in both the wild-type and Δ0928 strains, suggesting the 0928C strain has incomplete complementation of Lsp function. Immunoblots using antibodies to PiuA and lysates of a 50/50 mixture of the wild-type and Δ0928 strains produced two bands corresponding in size to those seen in the immunoblots of the separate wild-type and Δ0928 bacteria, confirming that the small differences in size in the proteins were not due to differences in running speed between lanes (data not shown).

### Lipoproteins in the Δ0928 strain are localized to the cell membrane

**Fig. 4:**
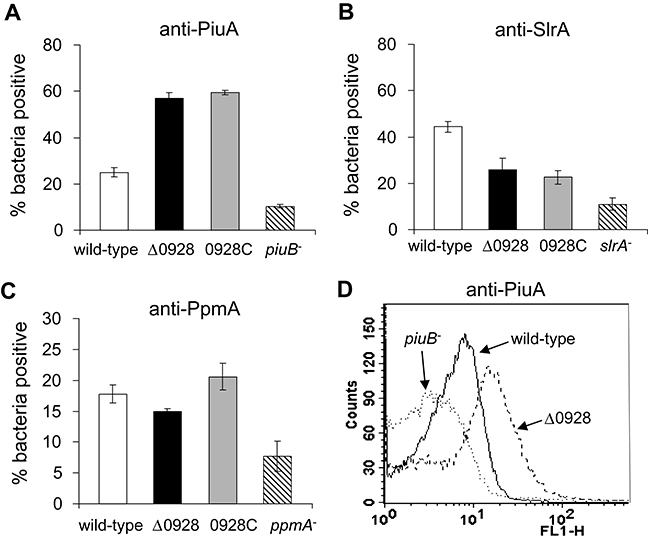
Flow cytometry analysis of the expression of the lipoproteins PiuA (A), SlrA (B) and PpmA (C) on the surface of live wild-type, Δ0928 and 0928C *S. pneumoniae* strains. As a control for non-specific binding of the antisera, strains deficient in expression of PiuA (*piuB*^–^, Brown *et al*., 2001a), PpmA or SlrA (*ppmA*^–^ and *slrA*^–^, Hermans *et al*., 2006) are included. Binding of anti-PiuA, anti-SlrA and anti-PpmA was significantly greater for the wild-type, Δ0928 and 0928C strains compared with the *piuB*^–^, *slrA*^–^ or *ppmA*^–^ mutant strains respectively (*P* < 0.05 for all strains, Student's *t*-test). Expression of PiuA was greater on the Δ0928 and 0928C strains compared with the wild-type (*P* < 0.01), and the expression of SlrA was less on the Δ0928 and 0928C strains compared with the wild type (*P* < 0.01). D. Example of flow cytometry histograms for the wild-type (black line), Δ0928 (dashed line) and *piuB*^–^ strains (dotted lines) probed with anti-PiuA.

**Fig. 5:**
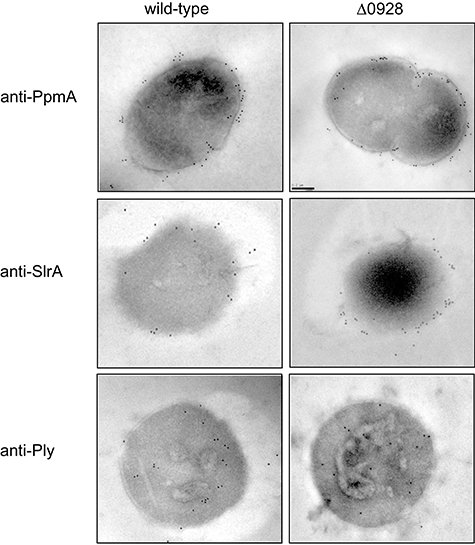
Immunoelectron microscopy of thin sections of the wild-type and Δ0928 *S. pneumoniae* strains using antibodies to the PpmA and SlrA lipoproteins. Controls with bacteria stained with the secondary antibody alone demonstrated only one or two gold particles per cell (not shown), and controls probed with anti-Ply are included to demonstrate the pattern of protein expression for an intracellular protein. The scale bar in the top right panel is 0.2 μm long.

The localization of lipoproteins in the Δ0928 strain was investigated using immunoblots of Triton X-114 extractions probed with antibodies to the *S. pneumoniae* lipoproteins. Triton X-114 is a non-ionic detergent which solubilizes and extracts integral membrane proteins into the detergent phase, with hydrophilic proteins partitioning into the aqueous phase (Bordier, 1981). For all three of the wild-type, Δ0928 and 0928C strains, the immunoblots demonstrated that all the lipoproteins investigated were in the detergent phase with no detectable signal in the aqueous phase, whereas for the negative control pneumolysin, there was no detectable protein in the detergent phase (Fig. 3). These results indicate that in the Δ0928 mutant strain, despite loss of Lsp function, the lipoproteins are still localized to the membrane. Surface accessibility of lipoproteins in the Δ0928 strain was confirmed using flow cytometry to assess the binding of antibodies to PiuA, SlrA and PpmA to live *S. pneumoniae* cells, using the *piuB*^–^, *slrA*^–^ and *ppmA*^–^ mutants (JSB3*SlrA*^–^ and *JSB3ppmA*^–^) strains as negative controls (Brown *et al*., 2001a; Hermans *et al*., 2006) (Fig. 4). Only very low levels of binding to each lipoprotein-deficient mutant strain were seen with the corresponding antibody, confirming the specificity of the assay. There was significant binding of antibodies to PiuA, SlrA and, at a lower level, PpmA in the wild-type, Δ0928 and 0928C strains, indicating that in all three strains, these lipoproteins are surface accessible. The level of binding of anti-PiuA was higher to both the Δ0928 and 0928C strains compared with the wild-type strain, whereas the level of binding of anti-SlrA was lower to both the Δ0928 and 0928C strains compared with the wild-type strain. To further demonstrate surface expression of lipoproteins in the Δ0928 strain, immunoelectron microscopy was performed using antibodies to the intracellular protein pneumolysin (as a control), and to PpmA and SlrA. Similar studies could not be done with anti-PiuA, as only limited expression of PiuA was detected using immunoelectron microscopy. All three proteins were detected, but there was a marked difference in the patterns of distribution between pneumolysin and the lipoproteins (Fig. 5). Pneumolysin was mainly detected within the cell, whereas SlrA and PpmA were detected almost entirely on the surface of the bacteria with no discernible differences in the distribution pattern identified between the wild-type and Δ0928 strains. Overall, the data obtained from the immunoblots of Triton X-114 extractions, flow cytometry and immunoelectron microscopy demonstrate that in the Δ0928 strain the unprocessed lipoproteins are still surface-expressed and attached to the cell membrane.

### Global analysis of *S. pneumoniae* lipoproteins in the Δ0928 strain

**Fig. 6:**
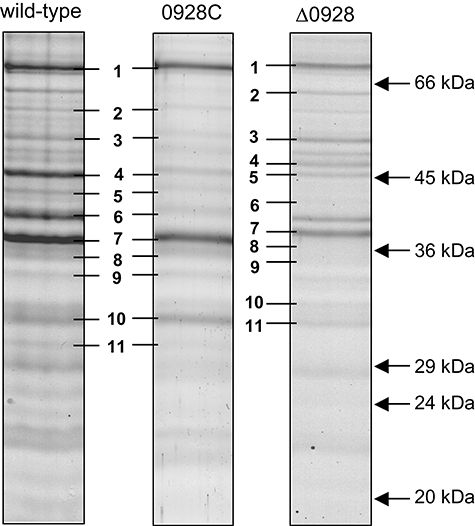
Coomassie stained SDS-PAGE of Triton X-114 extracts from wild-type, 0928C and Δ0928 *S. pneumoniae* strains. Numbered lines correspond to the band numbers shown in Table 2, and the gels are aligned so that the migration positions of molecular mass markers (marked for the Δ0928 strain gel) are identical between the three gels.

The band patterns of Coomassie stained gels of Triton X-114 extracts from the wild-type and Δ0928 strains were significantly different (Fig. 6), suggesting that there were major differences in lipoproteins between these strains. To precisely identify the proteins within these bands, they were excised, digested in gel using trypsin, and the digests analysed by liquid chromatography/tandem mass spectrometry. Proteins were identified by interrogation of the translated genomic database of *S. pneumoniae* strain TIGR4. Sixteen lipoproteins were identified in the Triton X-114 extracts from the wild-type and Δ0928 (Table 2), with a median of four (range 2–23) peptides sequenced per protein, resulting in a median coverage of the proteins' predicted amino acid sequence of 24% (range 7–65%) and a median probability of correct identification of the protein of 3.9 × 10^−7^ (range from 1.3 × 10^−3^ to 6.6 × 10^−13^). The estimated sizes of nearly all the identified lipoproteins were slightly larger than the sizes predicted from the derived amino acid sequence for the corresponding gene, probably reflecting the presence of lipid attached to the proteins in the Triton X-114 extracts (Table 2). There were clear differences between the wild-type and the Δ0928 strain in the estimated size of the majority of the lipoproteins (Fig. 6). In all cases except band 3 (Sp0092), these differences were an increase in the mass of the lipoprotein in the Δ0928 strain similar to the predicted increase in lipoprotein size if the N-terminal signal peptide was retained (Table 2). Complementation of strain Δ0928 restored the mass of each lipoprotein to that observed in the wild-type strain (Fig. 6). These data demonstrate that deletion of Sp0928 has global effects on lipoproteins compatible with loss of Lsp function.

**Table 2:** Identity according to LC-MS/MS data and estimated size of proteins present in the corresponding band number for the Triton X-114 extracts shown in Fig. 6 compared to the predicted sizes from sequence data for the processed lipoprotein and its N-terminal signal peptide.

Band^a^	Sp number^b^	Protein name	Predicted size of processed protein (kDa)	Predicted size of N-terminal signal peptide (kDa)	Estimated size in wild-type strain (kDa)	Estimated size in Δ0928 strain (kDa)
1	Sp1527	AliB	70.1	2.5	> 66	> 66
1	Sp 1891	AmiA	70.3	2.2	> 66	> 66
2	Sp2169	AdcA	54.3	1.9	59.2	61.8
3	Sp0092	Unknown function ABC transporter lipoprotein	52.1	2.4	54.4	53.2
4	Sp2108	MalX	42.9	2.4	47.8	50.0
5	Sp1683	Putative sugar ABC transporter lipoprotein	46.2	2.1	45.8	47.8
6	Sp1032	PiaA	35.3	2.2	43.0	45.8
7	Sp0845	Unknown function ABC transporter lipoprotein	34.6	2.1	39.5	41.2
7	Sp0981	PpmA	32.4	2.0	39.5	41.2
8	Sp1650	PsaA	32.6	2.0	37.8	38.6
9	Sp0149	Unknown function ABC transporter lipoprotein	29.0	2.2	34.0	36.2
9	Sp0771	SlrA	27.4	1.7	34.0	36.2
10	Sp1400	PstS	28.7	2.5	31.9	34.0
10	Sp0148	Unknown function ABC transporter lipoprotein	28.3	2.3	31.9	34.0
11	Sp1500	Putative amino acid-binding lipoprotein AatB	28.8	2.2	29.9	31.9
11	Sp0629	Conserved hypothetical protein	24.4	2.0	29.9	31.9

a Some bands contained more than one lipoprotein.

b Annotation for the corresponding gene within the TIGR4 genome (Tettelin *et al*., 2001).

### Analysis of phenotypes associated with ABC transporter functions in the Δ0928 strain

As lipoproteins form integral constituents of ABC transporters, we hypothesized that unprocessed lipoproteins in Δ0928 could lead to impaired ABC transporter function. We therefore analysed whether the Δ0928 strain had phenotypes similar to those previously associated with loss of function of some of the described *S. pneumoniae* ABC transporters.

**Fig. 7:**
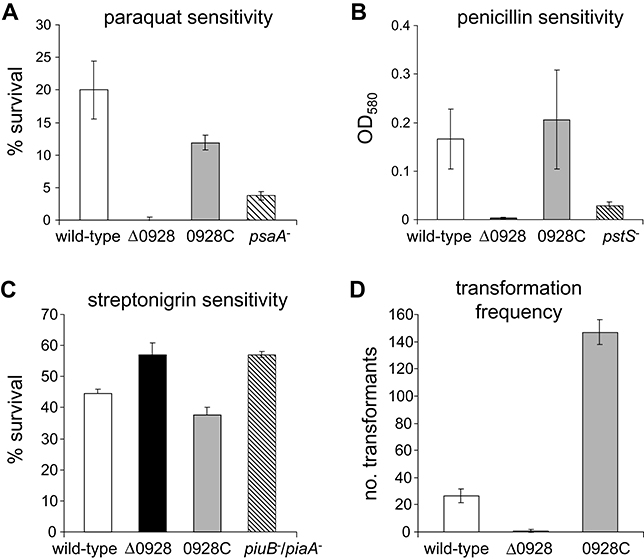
ABC transporter phenotype assays for the wild-type (clear columns), Δ0928 (black columns) and 0928C (grey columns) strains. A. Proportion of bacteria surviving incubation with 60 mM paraquat for 60 min, using the oxidative stress-sensitive *psaA*^–^ strain (diagonal shading) as a positive control. *P* < 0.01 (anova) for the Δ0928 and *psaA*^–^ strains versus the wild-type or 0928C strains. B. OD_580_ after incubation in THY broth containing 7.5 ng ml^−1^ penicillin for 10 h, using the penicillin-sensitive *pstS*^–^ strain (diagonal shading) as a positive control. *P* < 0.05 (anova) for the Δ0928 and *pstS*^–^ strains versus the wild-type or 0928C strains. C. Proportion of bacteria surviving incubation with 2 μg ml^−1^ streptonigrin for 30 min, using the partially streptonigrin-resistant *piuB*^–^*/piaA*^–^ strain (diagonal shading) as a positive control. *P* < 0.001 (anova) for the Δ0928 and *piuB*^–^*/piaA*^–^ strains versus the wild-type or 0928C strains. D. Number of kanamycin-resistant transformants obtained when transforming wild-type, Δ0928 and 0928C strains with genomic DNA from a kanamycin-resistant *S. pneumoniae* strain. *P* < 0.01 (anova) for the Δ0928 strain versus the wild-type or 0928C strains.

The *S. pneumoniae* manganese uptake ABC transporter PsaA is required for resistance to oxidative stress (Tseng *et al*., 2002; Chattopadhyay *et al*., 2003), and impaired *S. pneumoniae* ABC transporter function should lead to increased sensitivity to oxidative stress. Hence, sensitivity to oxidative stress of the wild-type, Δ0928 and 0928C strains was investigated using the redox compounds paraquat and hydrogen peroxide, using the *psaA*^–^ (JSB3*psaA*^–^) strain as a positive control (Hassett *et al*., 1987; Tseng *et al*., 2002). After exposure to 60 mM paraquat for 60 min, all the Δ0928 strain and 96% of the *psaA*^–^ strain had been killed, whereas the colony-forming units (cfu) of the wild-type and 0928C strains were 20% and 12%, respectively, of the inoculum (Fig. 7A). Similar results were obtained when the strains were exposed to 2 mM H_2_O_2_ (data not shown). These data show that the Δ0928 strain is more susceptible to oxidative stress.

Mutations affecting the phosphate uptake ABC transporter lipoprotein PstS result in increased sensitivity to penicillin (Soualhine *et al*., 2005). To test whether the Δ0928 strain was more sensitive to penicillin, the wild-type, Δ0928, 0928C and a *pstS*^–^ (JSB3*PstS*^–^, as a positive control) strains were cultured in Todd Hewitt Yeast (THY) broth in the presence of low concentrations of penicillin, and the extent of growth assessed by measuring the OD_580_ after 10 h of incubation. At a penicillin concentration of 7.5 ng ml^−1^, the OD_580_ for the Δ0928 and *pstS*^–^ strains were markedly lower compared with the wild-type and 0928C strains (Fig. 7B), demonstrating that the Δ0928 strain has increased sensitivity to penicillin.

Streptonigrin is an antibiotic which requires the presence of intracellular iron for its bactericidal action (Yeowell and White, 1982), and increased resistance to streptonigrin has been associated with mutations affecting the iron uptake ABC transporters Piu, Pia and Pit (Brown *et al*., 2001a; 2002). The effect of streptonigrin on the survival of the wild-type, Δ0928, 0928C and double-mutant *piuB*^–^/*piaA*^–^ (as a positive control) strains was studied by incubating the strains with 2 μg ml^−1^ streptonigrin and by determining the proportion of bacteria surviving by plating serial dilutions. After 30 min incubation, 55% of the Δ0928 strain survived, a similar proportion to the iron uptake ABC transporter double-mutant *piuB*^–^/*piaA*^–^ strain, but significantly greater than the proportion of the wild-type or 0928C strains surviving (less than 44%; Fig. 7C). These results suggest that the intracellular iron concentration was lower in the Δ0928 strain, and therefore that iron transport into the cell, which is mainly reliant on ABC transporters, may be partially impaired.

Zinc and manganese ions are a specific requirement for competence stimulating peptide (CSP)-induced *S. pneumoniae* transformation, and mutants affecting the zinc and manganese ABC transporters Adc and Psa respectively have reduced transformation efficiency (Dintilhac *et al*., 1997). To investigate whether the Δ0928 strain had a similar phenotype, a transformation efficacy assay was performed using genomic DNA from an *S. pneumoniae* strain carrying a kanamycin-resistant marker as donor DNA and the wild-type, Δ0928 and 0928C strains as parent strains. The Δ0928 strain was resistant to transformation, with very few transformants obtained, whereas transformation of both the wild-type and 0928C strains resulted in large numbers of transformants (Fig. 7D). Similarly, transformation of the Δ0928 strain was markedly impaired using *S. pneumoniae* genomic DNA carrying a chloramphenicol-resistant marker as donor DNA (data not shown).

Overall, the results of these assays demonstrate that the Δ0928 strain has several phenotypes associated with impaired ABC transporter function, suggesting that the loss of Lsp function in this strain has resulted in widespread effects on lipoprotein function.

### Growth of the Δ0928 strain in complete and stress media

**Fig. 8:**
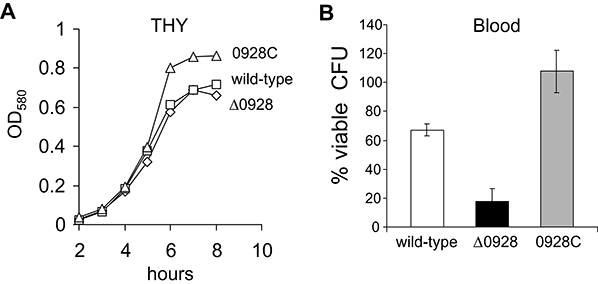
A. Growth of the wild type (squares), Δ0928 (diamonds) and 0928C (triangles) strains in THY broth measured by OD_580_. B. Bacterial cfu for the wild-type (clear columns), Δ0928 (black columns) and 0928C (grey columns) strains expressed as a proportion of the inoculum after incubation in human blood for 4 h. *P* < 0.01 (anova) for the Δ0928 strain versus the wild-type or 0928C strains.

Because of the wide range of cellular functions associated with different ABC transporters, global impairment of ABC transporter function might be predicted to inhibit growth of *S. pneumoniae*, especially under stress conditions such as cation-depleted or osmotic stress media. However, compared with the wild-type and 0928C strains, the growth of the Δ0928 strain was not impaired in an undefined nutrient-rich medium (THY extract) or in a chemically defined medium (CDM), and was only minimally impaired in THY treated with chelex−100 to remove cations (chelex-THY) or in THY containing 100 mM NaCl or 200 mM sucrose to create high osmotic stress conditions (Brown *et al*., 2001a; 2004; Fig. 8A and data not shown). However, *S. pneumoniae* is an obligate human commensal or pathogen and, therefore, THY does not adequately represent its natural environment. To investigate whether loss of Lsp function in the Δ0928 strain results in impaired growth under physiologically relevant conditions, freshly obtained human blood was inoculated with 10^6^ cfu ml^−1^ wild-type, Δ0928 and 0928C strains and bacterial cfu after 4 h incubation calculated by plating serial dilutions. For the Δ0928 strains, only 18% of the inoculum was viable after 4 h, compared with 67% and 108% for the wild-type and 0928C strains (Fig. 8B), demonstrating that loss of Lsp function results in impaired growth or survival in a physiologically relevant growth medium.

### Impaired virulence of the Δ0928 strain in mouse models of septicaemia and pneumonia

**Fig. 9:**
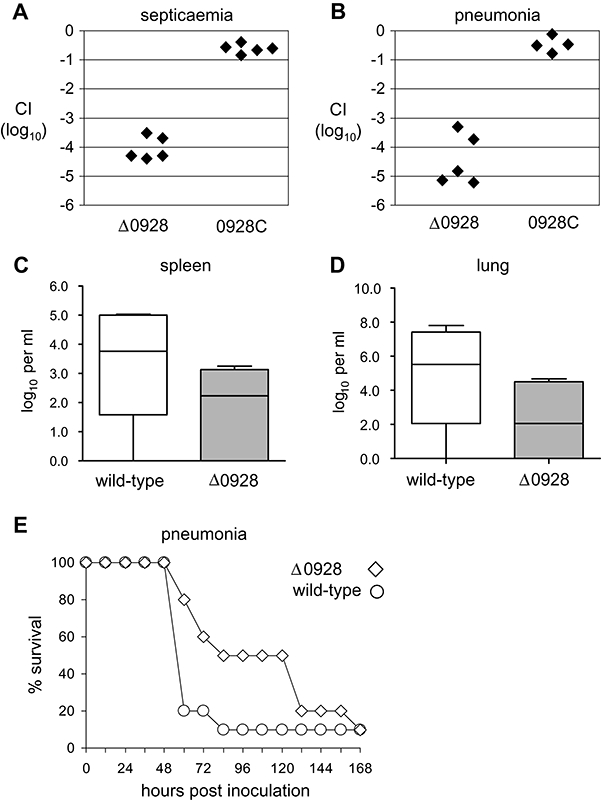
Assessment of the virulence of the Δ0928 mutant strain. A and B. CIs (expressed as log_10_) for the Δ0928 and 0928C strains versus the wild-type strain in mouse models of septicaemia (A) and pneumonia (B). Each point represents the CI for a single animal, and for both models, *P* < 0.001 for the comparison of the CIs for the Δ0928 and 0928C strains (Students *t*-test). C and D. Log_10_ ml^−1^ bacterial cfu recovered from the spleen (C) and lungs (D) from groups of six mice 42 h after inoculation i.n. with 10^6^ cfu of the wild-type and Δ0928 strains. Results are shown as box and whisker diagrams of the median cfu ml^−1^ and the interquartile range. For the comparisons of the results for the Δ0928 strain versus the wild-type strain for spleens and lungs *P* = 0.047 and 0.033 (Mann–Whitney *U*-test) respectively. D. Time-course of the development of fatal infection for groups of 10 mice inoculated i.n. with 10^6^ cfu of the wild-type and Δ0928 strains (*P* = 0.14, log rank test).

The impaired survival of the Δ0928 strain in blood suggested that this strain might be attenuated in virulence in infection models. Hence, the virulence of the Δ0928 and 0928C strains were compared with the wild-type strain using mixed infections and the competitive index (CI) in mouse models of both septicaemia and pneumonia. In both models for the comparison of the wild-type with the Δ0928 strain, the CI was markedly reduced at 0.00013 ± 0.00012 24 h after intraperitoneal (i.p.) inoculation, and at 0.00001 ± 0.000004 48 h after intranasal (i.n.) inoculation (Fig. 9A and B). In contrast, mixed infections of the 0928C strain and the wild-type strain demonstrated that the complemented strain was only slightly reduced in virulence (CIs of 0.61 ± 0.16 and 0.47 ± 0.27 after i.p. or i.n. inoculation respectively), confirming that the loss of virulence of the Δ0928 strain was due to deletion of *lsp*.

To further analyse the effects of loss of Lsp function on virulence, bacterial cfu in the lungs and spleen 42 h after inoculation (the time point just before fatal infection has developed in mice inoculated with the wild-type *S. pneumoniae* strain) and the course of *S. pneumoniae* pulmonary infection over time were assessed in groups of mice given a pure inoculum i.n. of the wild-type or the Δ0928 strain. For mice infected with the Δ0928 strain, *S. pneumoniae* cfu in the lungs and spleen were 3.5 and 1.5 log_10_ lower 42 h after inoculation compared with mice infected with the wild-type strain (Fig. 9C and D). In the time-course infection experiments, the majority of mice inoculated with the wild-type strain developed fatal infection within 60 h, whereas by this time point, no mice infected with Δ0928 had developed fatal infection (Fig. 9E). However, by 168 h, lethal infection had developed in 90% of mice infected with the Δ0928 strain. Bacteria recovered from mice developing fatal infection after inoculation with the Δ0928 strain were still resistant to erythromycin, demonstrating that these bacteria had not lost the *lsp* mutation. Overall, these data demonstrate that although the development of infection with the Δ0928 strain was significantly delayed, this strain was still capable of causing lethal infection.

## Discussion

The importance of different lipoproteins for *S. pneumoniae* virulence and the identification of an operon containing the putative *S. pneumoniae lsp* gene by STM screens prompted our investigation of the function and the role of Lsp in *S. pneumoniae.* The predicted protein encoded by the putative *S. pneumoniae lsp* gene Sp0928 has high levels of identity to Lsp from a variety of bacteria, as well as identical conserved amino acids within functional domains and the same predicted transmembrane profile as the *B. subtilis* Lsp. Expression of Sp0928 in *E. coli* protected against globomycin, a cyclic peptide antibiotic that is an antagonist of Lsp (Inukai *et al*., 1978), and immunoblots and proteomic analysis of lipoproteins demonstrated that in the Δ0928 strain the majority of the identified lipoproteins have a slightly higher molecular weight consistent with failure of cleavage of the N-terminal signal peptide. Although absolute confirmation that Sp0928 encodes an Lsp would require sequencing the N terminus of a range of lipoproteins from the Δ0928 strain, our data strongly support the results of the blast searches suggesting that Sp0928 encodes the *S. pneumoniae* Lsp, and is essential for the processing of lipoproteins to the mature form.

The *S. pneumoniae* genome contains over 40 genes that are predicted to encode lipoproteins (Bergmann and Hammerschmidt, 2006), which existing data and by analogy to described ABC transporters of other species suggest, will contribute to a wide variety of cellular functions important for growth and replication. The effects of complete loss of function of all these lipoproteins therefore would be expected to have profound effects on *S. pneumoniae*. However, despite changes in the size of at least 13 lipoproteins, the *lsp* mutant strain had normal growth in laboratory media, and only minimal growth delay in stress media has been previously reported to affect growth of *S. pneumoniae* strains with mutations in specific ABC transporters (Brown *et al*., 2001a). These results suggest that although loss of Lsp function resulted in impaired processing of lipoproteins, these lipoproteins still function adequately for the *lsp* mutant strain to grow normally under the *in vitro* conditions investigated. Investigation of particular phenotypes associated with the function of specific ABC transporters (sensitivity to oxidative stress, streptonigrin and penicillin, and transformation frequency) demonstrated that these are all impaired in the *S. pneumoniae lsp*-deletion strain, but not in the complemented strain, confirming that they were due to loss of *lsp* rather than other effects in the mutated strain. Hence, the *lsp* mutant strain does have widespread defects in ABC transporter function, although these are not strong enough to affect growth in laboratory media. This phenotype is broadly similar to that described for *lsp* mutant strains of other Gram-positive bacteria, which contain unprocessed prolipoprotein but, in contrast to *E. coli*, still grow well in laboratory media (Isaki *et al*., 1990; Sankaran and Wu, 1995; Tjalsma *et al*., 1999a; De Greef *et al*., 2003; Reglier-Poupet *et al*., 2003; Venema *et al*., 2003). However, in contrast to our results, mutation of the *B. subtilis lsp* impairs growth under stress conditions without affecting lipoprotein-dependent functions like genetic competence, sporulation and germination (Tjalsma *et al*., 1999a). These data as well as the other differences between the phenotypes of *lsp* mutants in different bacterial species discussed below suggest that the effects of loss of *lsp* on bacterial phenotypes in different Gram-positive species cannot be easily predicted. For some assays, the complemented *lsp*-deletion strain had improved phenotypes compared with the wild-type strain (growth in blood or THY, and transformation frequency). The reasons for these observations are not fully understood, but probably reflect the reduced expression of Sp0929 and *cbpE* and the increased expression of Sp0927 and *lsp* in the complemented 0928C strain. These differences in gene transcription for the Sp0927–0930 operon may also explain why the 0928C strain is not fully virulent, with a CI of 0.61 and 0.47 in the septicaemia and pneumonia models respectively.

There are several mechanisms by which lipoprotein function could be partially preserved in the *S. pneumoniae lsp* mutant strain. Alternative processing pathways for lipoproteins have been suggested for *B. subtilis* and *S. suis* (Tjalsma *et al*., 1999a; De Greeff *et al*., 2003), and these could potentially limit the effects of loss of Lsp function. However, in the *S. pneumoniae lsp* mutant strain, processed lipoprotein forms were possibly present only for the non-ABC transporter lipoproteins, PpmA and SlrA, and alternative processing of lipoproteins is therefore unlikely to account for the almost normal growth of the *lsp* mutant strain in laboratory media. Although recent evidence suggests that Lsp activity in *L. monocytogenes* does not require Lgt (Baumgärtner *et al*., 2007), for most eubacteria lipid attachment of prolipoproteins has generally been accepted to occur before Lsp activity (Tokunaga *et al*., 1982; Sankaran and Wu, 1995; Sutcliff and Harrington, 2002). Using antibodies to lipoproteins and immunoblots of Triton X-114 extracts, flow cytometry assays and electron microscopy, we have demonstrated that *S. pneumoniae* follows this general model of lipoprotein processing, with lipoproteins still present in the detergent phase and expressed on the bacterial surface in the *S. pneumoniae lsp* mutant strain. Hence, despite incomplete processing, lipoproteins in the *S. pneumoniae lsp* mutant strain are still attached to cell membranes and could remain at least partly functional. The flow cytometry data also suggested that there was a higher level of expression of PiuA in the *lsp* mutant strain, and overexpression of ABC transporters may provide some compensation for the impaired function of unprocessed lipoproteins. In addition, functions of individual ABC transporters are frequently redundant and shared with non-ABC transporter import systems (e.g. sugar transport; Tettelin *et al*., 2001), and this could limit the effects on growth of loss of Lsp function. Full evaluation of any compensatory mechanisms for loss of Lsp function in the *S. pneumoniae lsp* mutant strain will provide a better understanding of the importance of different lipoprotein functions and requires further investigation.

Although the *S. pneumoniae lsp* mutant strain grew well in laboratory media, the growth of this strain was significantly impaired in blood compared with the wild-type strain. Growth in blood is likely to cause markedly more physiological stress (e.g. through nutrient and mineral limitation, and oxidative and osmotic stress) on *S. pneumoniae* than growth in complete laboratory media. Partial impairment of ABC transporter and non-ABC transporter lipoprotein functions under these conditions could therefore lead to significant growth or survival defects. The impaired survival and/or growth of the *lsp* mutant strain in blood was reflected in the results of the virulence assays, which demonstrated a markedly impaired CI for the *lsp* mutant strain in mouse models of both the pneumonia and septicaemia, and recovery of fewer bacteria from target organs 48 h after i.n. inoculation compared with the wild-type strain. However, the majority of mice infected with the *lsp* mutant strain did develop fatal infection, indicating that the *lsp* mutant strain has delayed replication during infection rather than an inability to cause fatal disease. The cumulative effects of total loss of function of the numerous lipoproteins and ABC transporters required for virulence (Dintilhac *et al*., 1997; Polissi *et al*., 1998; Brown *et al*., 2001a; 2002; Lau *et al*., 2001; Hava and Camilli, 2002; Hermans *et al*., 2006) should have a profound effect on virulence. This is illustrated by dual mutation of the Piu and Pia iron uptake ABC transporters alone or deletion of *lgt*, possibly leading to failure to retain lipoproteins on the cell membrane, both of which completely abrogate *S. pneumoniae* virulence in mice models of pneumonia (Brown *et al*., 2001a; Petit *et al*., 2001). Therefore, the ability of the *S. pneumoniae lsp* mutant to still cause fatal disease reinforces that there is only partial impairment of ABC transporter and lipoprotein function in this strain. Deletion of *lsp* also affects the virulence of the Gram-positive pathogens *L. monocytogenes* and *M. tuberculosis* (Reglier-Poupet *et al*., 2003; Sander *et al*., 2004), but not *S. suis* (De Greeff *et al*., 2003). Why loss of Lsp does not affect the virulence of *S. suis* but has a significant effect on *in vivo* replication of its close relative *S. pneumoniae* requires further evaluation. Furthermore, in contrast to the results for *S. pneumoniae*, mutation of the *S. aureus lgt* actually enhances virulence, despite leading to loss of lipoproteins from the bacterial surface (Wardenburg *et al*., 2006). This is thought to be due to the loss of surface lipoproteins reducing recognition of the mutant strain by Toll-like receptors (Stoll *et al*., 2005; Wardenburg *et al*., 2006).

To summarize, we have confirmed that Sp0928 encodes the *S. pneumoniae* Lsp and described the phenotype of a strain in which this gene has been deleted. The data show that in *S. pneumoniae* Lsp is required for cleavage of the lipoprotein signal peptide sequence after attachment to the cell membrane, and that loss of Lsp leads to accumulation of immature lipoproteins, impaired ABC transporter function and reduced *S. pneumoniae* replication in animal models of infection. It is likely that Lsp will be important for disease pathogenesis of other bacterial pathogens, and therefore may offer a potential target for novel therapeutic agents. However, there are significant differences in the effects of loss of *lsp* between even relatively closely related bacterial species, and the effects of deletion of *lsp* on the *in vitro* and *in vivo* phenotypes of *S. pneumoniae* indicate that lipoprotein function is only partially affected. Further research is necessary to clarify the roles of Lsp in different bacteria, and the mechanisms by which *S. pneumoniae* can compensate for loss of Lsp function.

## Experimental procedures

### Bacterial strains and culture conditions

*Streptococcus pneumoniae* strains used in this work are listed in Table 3. The mutant strains used for this work were constructed in the 0100993 capsular serotype 3 clinical isolate (Lau *et al*., 2001). *S. pneumoniae* strains were cultured at 37°C and 5% CO_2_ on Columbia agar supplemented with 5% horse blood, in THY or CDM (Samen *et al*., 2004). Chloramphenicol (10 μg ml^−1^), erythromycin (0.2 μg ml^−1^), spectinomycin (200 μg ml^−1^) and kanamycin (1 mg ml^−1^) were added to blood agar plates where appropriate. Cations were depleted from the THY medium using 2% chelex-100 (Bio-Rad) and THY NaCl as previously described (Brown *et al*., 2001a; 2004). The growth of different strains were compared in broth culture by measuring OD_580_ at hourly intervals. Working stocks of bacterial cultures in THY (OD_580_ 0.3–0.4) were stored at −80°C with 10% glycerol. Plasmids were amplified in *E. coli* strain DH5α, grown at 37°C on Luria–Bertani (LB) medium with appropriate selection (Sambrook *et al*., 1989).

**Table 3:** Strains, plasmids and primers constructed and used in this study.

Name	Description/sequence (source/reference)
Strains	
0100993	*S. pneumoniae* capsular serotype 3 clinical isolate (Lau *et al*., 2001)
Δ0928	0100993 with in-frame deletion of Sp0928: ery^r^ (this study)
0928C	Δ0928 with in-frame replacement of *erm* with Sp0928 and *cat*: cm^r^ (this study)
*piuB*^–^*/piaA*^–^	0100993 containing an insertion in *piuB* and in *piaA*: ery^r^ cm^r^ (Brown *et al*., 2001a)
TIGR4-*psaA*^–^	*S. pneumoniae* TIGR4 strain with mariner mutation in *psaA*: spec^r^ (McCluskey *et al*., 2004)
JSB3*psaA*^–^	0100993 with mariner mutation in *psaA*: spec^r^ (this study)
JSB3*pstS*^–^	0100993 containing chromosomal insertion of pHS102: cm^r^ (this study)
D39Δ*slrA*	*S. pneumoniae* D39 strain with deletion of *slrA*: ery^r^ (Hermans *et al*., 2006)
JSB3*slrA*^–^	0100993 with deletion of *slrA*: ery^r^ (this study)
JSB3*ppmA*^–^	0100993 with deletion of *ppmA*: ery^r^ (this study)
Plasmids	
pPC143	pQE30UA carrying full-length Sp0928 from 0100993: Km^r^, Amp^r^ (this study)
pPC138	pQE30UA carrying full-length Sp0149 from 0100993: Km^r^, Amp^r^ (this study)
pHS102	Disruption vector for the *S. pneumoniae pstS* gene: cm^r^ (Soualhine *et al*., 2005)
Primers	
Sp0926.1	GCCTTCTGGGATGTGTGG
Sp0927.1	GGAAGTTCTTATAGTCAG
Sp0927.2	CCACCAACTATTACGGCC
Sp0928.1	CAGTGGAATCTGCTGGAC
Sp0928.2	GATTGGGCTGGATCAGTTG
Sp0929.1	CGACATCACCCTCCTGGAC
Sp0929.2	GGCTGTAACTGCTAAAGG
Sp0930.1	GATCACTGTGGGTATGGG
Sp0930.2	GTGGTGCCTGGTTAGTGG
Sp0931.1	CGACAAAGCCTGATGGGC
Sp0927F	GCTAGCCAGCACTATGAC
Ery-Sp0927R	TATTTTATATTTTTGTTCATGATTTCCTCTTTTGATCAAAATA
Sp0928F	GGACGCCTATGCGACAGG
Sp0928R	CGGAAGATCCTCAGCCAC
Ery-Sp0929F	ATTATTTAACGGGAGGAAATAAATGGAAATTAAAATTGAAACTGG
SP0929R	GCGCCCTGTCTCCAGTTG
Sp0930F	ATGAAAAAGAAATTAACTAGT
Sp0930R	CCAATGAGAGGACCATAC
Sp0928F-comp	ATTATTTAACGGGAGGAAATAAATGAAAAAAAGAGCAATAGTGGCAG
Cm-Sp0928R	GCCTAATGACTGGCTTTTATAATTTAATTTCCATTTATTTCCTC
Cm-Sp0929F	ACATTATCCATTAAAAATCAAAATGGAAATTAAAATTGAAACTGG
Sp0928F-Exp	ATGAAAAAAAGAGCAATAGTGGCAG
Sp0928R-Exp	GTAATCACTCTTAATTTCCATTTATTTCCTCTT
EryF	ATGAACAAAAATATAAAATA
EryR	TTATTTCCTCCCGTTAAATAAT
CmF	TTATAAAAGCCAGTCATTAG
CmR	TTTGATTTTTAATGGATAATG
RT-16SrRNAF	TGAAGAAGGTTTTCGGATCG
RT-16SrRNAR	CGCTCGGGACCTACGTATTA
RT-Sp0927F	CGCAAGGGCATAGTGAGATT
RT-Sp0927R	GGATGACCCTCACGGAGATA
RT-Sp0928F	GCAGCTGTTATTCGCTGTCA
RT-Sp0928R	GACCACCCGCGATTATTAGA
RT-Sp0929F	CGGGTATCAATGGGGTTCT
RT-Sp0929R	CCCAATATTTGCGGAGAGAC
RT-Sp0930F	CCTATCCAGTTGACCGAGTCT
RT-Sp0930R	CACCTTTTTCTGCAGCAGTC

### Construction of mutant strains

For the in-frame deletion of Sp0928, a construct was created in which 784 bp of flanking DNA 5′ to the Sp0928 ATG (primers Sp0927F and Ery-Sp0927R) and 669 bp of flanking DNA 3′ to the Sp0928 ORF (starting from the ATG of the overlapping Sp0929 ORF, primers Ery-Sp0929F and Sp0929R) were amplified by PCR and fused with the *erm* cassette from pACH74 (a suicide vector carrying *erm* for selection in *S. pneumoniae*, a kind gift from J. Paton) by overlap extension PCR (Link *et al*., 1997). For complementation of the Δ0928 strain, a construct was made using overlap extension PCR that consisted of the following PCR products: the full-length Sp0928 ORF and 626 bp of Sp0927 (primers Sp0927F and Cm-Sp0928R); the chloramphenicol-resistance marker (*cat*, primers CmF and CmR); and 677 bp sequence 3′ to Sp0928 starting at the ATG codon of Sp0929 (primers Cm-Sp0929F and Sp0929R). Primers used for the overlap extension PCRs are shown in Table 3. These constructs were transformed into *S. pneumoniae* by homologous recombination and allelic replacement using CSP-1 and standard protocols (Havarstein *et al*., 1995; Lau *et al*., 2001). The identity and accuracy of the mutations were confirmed by PCR analysis and sequencing. Both the Δ0928 and 0928C strains were stable after two 8 h growth cycles in THY without antibiotic. The JSB3*psaA*^–^, JSB3*ppmA*^–^ and JSB3*slrA*^–^ mutant strains in the 0100993 background were constructed by transformation with genomic DNA from existing *psaA*^–^, *ppmA*^–^ and *slrA*^–^ (kind gift from Peter Hermans; McCluskey *et al*., 2004; Hermans *et al*., 2006), and the JSB3*pstS*^–^ mutant strain using the *pstS* disruption vector pHS102 (kind gift from Marc Ouellette; Soualhine *et al*., 2005).

### DNA, RNA extraction and RT PCR

Genomic DNA and total RNA were isolated from *S. pneumoniae* strains using the Wizard genomic DNA isolation kit and the SV total RNA isolation system (Promega) respectively, following the manufacturer's instructions except that cells were incubated with 0.1% deoxycholic acid (Sigma) at 37°C for 10 min before extraction. 0.5% RNasin (Promega) was added to extracted RNA to prevent it from degradation. cDNA was derived and amplified from RNA using the Access RT-PCR system (Promega) and target-specific primers. The following primer pairs were used for the transcriptional analysis of the Sp0927–0930 operon structure: Sp0926.1 and Sp0927.1 for the junction between Sp0926 and Sp0927; Sp0927.2 and Sp0928.1 for Sp0927 and Sp0928; Sp0928.2 and Sp0929.1 for Sp0928 and Sp0929; Sp0929.2 and Sp0930.1 for Sp0929 and Sp0930; Sp0930.2 and Sp0931.1 for the junctions between Sp0930 and Sp0931 (Table 3). DNA contamination of all RNA samples was excluded by RT-PCR with no reverse transcriptase failing to result in a product.

### Real-time RT-PCR

cDNA was derived and amplified from 1 μg of total RNA in a 20 μl reaction volume using the Applied Biosystems Geneamp RNA PCR core kit (Applied Biosystems, UK) according to the manufacturer's instructions. Target-specific primers used for the analysis of gene expression of individual genes within the Sp0927–0930 operon are described in Table 3 (primers prefaced by RT-). Real-time RT-PCR was conducted using the Platinum SYBR Green qPCR SuperMix UDG (Invitrogen, UK) on a LightCycler 1.5 Real-time Detection System (Roche, UK), and analysed using LightCycler Real-time PCR Detection System Software Version 3.5. Cycling conditions were as follows: one cycle of 50°C (2 min), 95°C (2 min); 45 cycles of 95°C (5 s), 55°C (5 s), 72°C (15 s). The specificity of the PCR product was confirmed by melting curve analysis and gel electrophoresis. For each gene, crossing point (Cp) values were determined from the linear region of the amplification plot and normalized by subtraction of the Cp value for 16S RNA generating a ΔCp value. Relative change was determined by subtraction of the ΔCp value for the wild-type strain from the ΔCp value for the mutant strain (ΔΔCp value), and fold change calculated using the formula 2^−ΔΔCp^.

### Globomycin-resistance assay

Stationary phase *E. coli* cells were diluted 1:50 in LB broth-containing kanamycin (25 mg ml^−1^) and ampicillin (100 mg ml^−1^) grown for 5 h. These exponentially growing cells were inoculated (5%) in fresh LB containing different concentrations of globomycin (kind gift from Masatoshi Inukai, International University of Health and Welfare, Tochigi, Japan). The growth was then assessed by OD_600_ measurement after 16 h growth at 37°C and 220 rotations per min.

### Protein immunoblots and Triton X-114 extraction

Protein samples from whole-cell lysates and Triton X-114 extracts were separated on SDS-PAGE 10% and 15% resolving gels respectively, blotted onto nitrocellulose membranes and probed with specific antisera (1:2500 dilution) according to standard protocols (Sambrook *et al*., 1989). Membrane proteins were extracted by Triton X-114 extraction according to Bordier (1981) with modifications described by Cockayne *et al*. (1998). Briefly, exponentially growing *S. pneumoniae* cells were pelleted and digested with 100 μl of 0.1% DOC (Sigma) in PBS for 30 min at 37°C. The digested pellets were sonicated with three pulses of 15 s with a 10 s cooling time using a Soniprep 150 (Sanyo) ultrasonicator. Eight hundred microlitres of PBS and 100 μl of Triton X-114 (10% in PBS) were then added to the lysates, which were incubated at 4°C for 2 h, followed by centrifugation at 13 000 *g* for 10 min at 4°C to pellet insoluble debris. Supernatants were then incubated at 37°C for 30 min to allow phase separation, followed by centrifugation at room temperature to pellet the detergent phase proteins. The detergent phase pellet was then washed with 1 ml of PBS at 4°C for 1 h, followed by incubation at 37°C for 30 min and centrifugation to pellet the proteins. The detergent phase proteins were diluted 1:2 in PBS prior to solubilization in Laemmli sample buffer for SDS-PAGE.

### Global analysis of lipoproteins

For proteomic studies, wild-type and mutant strains were cultured in Brain Heart Infusion broth (Oxoid) until late exponential phase. Lipoproteins were extracted by treatment of washed cell suspensions with Triton X-114 as above except that cells were treated with mutanolysin and lysozyme. Extracts were analysed by SDS-PAGE (10% acrylamide), and gels were stained with Colloidal Coomassie brilliant blue (Sigma). Bands were excised from gels, and proteins were digested in gel with sequencing grade trypsin (Promega) (Wilkins *et al*., 2001). Tryptic digests were analysed by LC-MS/MS using a ProteomeX system (Thermo Scientific, Hemel Hempstead, Hertfordshire, UK). Chromatography was performed on a 100 × 0.18 mm BioBasic C_18_ column (Thermo Scientific). Peptides were eluted with aqueous ACN (5–80% ACN over 90 min) containing 0.1% formic acid at a flow rate of 2 μl min^−1^. Spectra were acquired in data-dependent MS/MS mode with dynamic exclusion prior to analysis using TurboSEQUEST software (Bioworks version 3.2; Thermo Scientific). Lipoproteins were identified using MS/MS data to interrogate the translated genomic sequence of the *S. pneumoniae* strains TIGR4, D39 and R6 (the J. Craig Venter Institute, JCVI; http://www.jcvi.org/). MS/MS spectra were required to have XCorr values of at least 1.5, 2.0 and 2.5 for peptides with a single, double or triple charge respectively, in order for these to be included in analyses.

### Lipoprotein expression analysis

Flow cytometry assays for the binding of polyclonal antiserum to the lipoproteins PiuA and SlrA on live *S. pneumoniae* were performed using a previously described protocol (Jomaa *et al*., 2005), affinity purified polyclonal antiserum to PiuA, PpmA and SlrA (1:50 dilution in PBS), and a 1:100 dilution of phycoerythrin-conjugated goat anti-rabbit (Sigma). After incubation on ice for 30 min, the bacteria were washed with 500 μl of PBS-0.1% Tween 20 and resuspended in 400 μl of PBS for flow cytometry analysis. Results are presented as the percentage of bacteria from each species bound with antibodies that specifically bind to the corresponding lipoproteins, using *psaA*^–^, *ppmA*^–^ and *slrA*^–^ strains as negative controls.

### Immunoelectron microscopy

Immunoelectron microscopy of *S. pneumoniae* was performed as previously described (Overweg *et al*., 2000). *S. pneumoniae* cells (10^8^ cfu) were fixed in a mixture of 4% formaldehyde and 0.1% glutaraldehyde, embedded in low-melting agarose (Sigma) and dehydrated in a graded series of methanol solutions (25–100%). They were then embedded in lowicryl K4M resin (Agar Scienfic, UK) with UV polymerization, and ultra-thin sections were transferred to Formvar-coated copper grids. The immunostaining was performed as described by Overweg *et al*. (2000) and examined with a JEOL JEM-1010 transmission electron microscope at 80 kV.

### ABC transporter phenotype analysis

Sensitivity to oxidative stress and cation transport were studied by exposure of *S. pneumoniae* strains (10^6^ cfu) to 60 mM paraquat (Sigma; Tseng *et al*., 2002) or 2 μg ml^−1^ streptonigrin (Sigma; Brown *et al*., 2001a) at 37°C for 60 and 30 min respectively. The proportion of survivors after the exposure was calculated by plating serial dilutions on blood agar plates. For penicillin sensitivity, 10^6^ cfu of exponentially growing *S. pneumoniae* cells were diluted in THY broth containing 7.5 ng ml^−1^ penicillin (Sigma), and the extent of growth was assessed by OD_580_ measurements after incubation at 37°C and 5% CO_2_ for 10 h. To compare transformation frequency, test strains at an OD_580_ of 0.015 were transformed using a standard transformation protocol (Lau *et al*., 2001) with 5 ng of *S. pneumoniae* genomic DNA containing a marker for kanamycin or chloramphenicol resistance.

### *In vivo* studies

All animal experiments conformed to institutional and governmental guidelines and regulations. Outbred CD1 female white mice (Charles Rivers Breeders) weighing 18–22 g were used for animal infection experiments. For the pneumonia model, mice were anaesthetized by inhalation of halothane (Zeneca) and inoculated i.n. with an inoculum volume of 50 μl, and for the septicaemia model by i.p. inoculation of an inoculum of 100 μl. Mixed infection experiments were used to calculate CIs (the ratio of mutant to wild-type strain recovered from the mice divided by the ratio of mutant to wild-type strain in the inoculum), using 5 × 10^6^ cfu each of the wild-type and mutant strains for the pneumonia model, and 4 × 10^3^ cfu each in 100 μl for the septicaemia model and groups of four or five mice. Mice were sacrificed after 24 h (septicaemia model) or 48 h (pneumonia model), target organs recovered and homogenized in 0.5 ml of 0.9% saline, before plating dilutions on non-selective and selective medium for calculation of the CI. To compare the course of disease between the Δ0928 and wild-type strains, groups of 10 mice were inoculated with 10^6^ cfu i.n. of each strain and closely observed over the next 14 days. Mice were sacrificed when they exhibited the following signs of disease: hunched posture, poor mobility, weight loss, coughing and tachypnoea. Additional groups of six mice were sacrificed 42 h after inoculation for calculation of bacterial cfu within the lungs and spleens by plating serial dilutions of organ homogenates.

### Statistical analysis of results

All *in vitro* data use three or more samples per strain tested, and are representative of experiments repeated at least twice that gave similar results. Results for phenotype assays were compared between strains using Student's *t*-test or anova. CIs for the Δ0928 and 0928C strains were compared using Student's *t*-test, and bacterial cfu from target organs compared using the Mann–Whitney *U*-test. Experiments comparing the course of disease between the Δ0928 and wild-type strains were repeated twice, giving similar results, and the data were analysed using the log rank method.
